# A Preformed Binding Interface in the Unbound Ensemble of an Intrinsically Disordered Protein: Evidence from Molecular Simulations

**DOI:** 10.1371/journal.pcbi.1002605

**Published:** 2012-07-19

**Authors:** Michael Knott, Robert B. Best

**Affiliations:** University of Cambridge, Department of Chemistry, Cambridge, United Kingdom; University of California Davis, United States of America

## Abstract

Intrinsically disordered proteins play an important role in cellular signalling, mediated by their interactions with other biomolecules. A key question concerns the nature of their binding mechanism, and whether the bound structure is induced only by proximity to the binding partner. This is difficult to answer through experiment alone because of the very heterogeneous nature of the unbound ensemble, and the probable rapid interconversion of the various unbound structures. Here we report the most extensive set of simulations on NCBD to date: we use large-scale replica exchange molecular dynamics to explore the unbound state. An important feature of the study is the use of an atomistic force field that has been parametrised against experimental data for weakly structured peptides, together with an accurate explicit water model. Neither the force field nor the starting conformations are biased towards a particular structure. The regions of NCBD that have high helical propensity in the simulations correspond closely to helices in the ‘core’ unbound conformation determined by NMR, although no single member of the simulated unbound ensemble closely resembles the core conformation, or either of the two known bound conformations. We have validated the results against NMR spectroscopy and SAXS measurements, obtaining reasonable agreement. The two helices which most stabilise the binding of NCBD with ACTR are formed readily; the third helix, which is less important for binding but is involved in most of the intraprotein contacts of NCBD in the bound conformation, is formed more rarely, and tends not to coexist with the other helices. These results support a mechanism by which NCBD gains the advantages of disorder, while forming binding-competent structures in the unbound state. We obtain support for this mechanism from coarse-grained simulations of NCBD with, and without, its binding partner.

## Author Summary

While many proteins have a specific ‘native’ conformation, so-called intrinsically disordered proteins (IDPs) adopt many different conformations in rapid succession—a characteristic that may be advantageous for rapid binding and promiscuous association. However, this characteristic also makes it very hard to make experimental measurements over times that are short enough to see changes of conformation. In this work, we use the results of a large-scale molecular simulation to explore conformations of NCBD, which is an IDP that adopts specific conformations when it binds either of two other proteins (ACTR and IRF-3). Our results point to the following hypothesis: to help NCBD bind ACTR, those parts of NCBD that make contact with it in the bound conformation are biased towards that conformation, even in the absence of ACTR. Other parts of NCBD tend to avoid the ACTR-bound conformation, to help ensure that NCBD is disordered when unbound.

## Introduction

We must consider all the conformations that a protein populates, if we want to understand completely its function and behaviour. Although many proteins fold promptly to a native conformation—thereby allowing us to use a simplified picture of conformational space—many others do not [1].

Intrinsically disordered proteins (IDPs) [2], [3], [4], [5] do not form stable structures in isolation under physiological conditions. Instead, they sample multiple conformations, often while retaining some residual structure [6]. An IDP may perform a function while partly disordered [7], [8], or it may fold to one or more ordered conformations as it binds ligands [9] in a process known as coupled folding and binding [10]; indeed IDPs are often signalling molecules, for example in transcription regulation [11]. Protein disorder has a number of possible advantages: for example, a larger effective binding surface may increase binding rates (via ‘fly-casting’ [12] or ‘non-native steering’ [13]), while greater conformational flexibility might help a protein to bind multiple ligands [2], [14], [15]. It has been proposed that disorder can aid allosteric coupling [16], enable ultrasensitivity [17] or, in contrast, help to stabilise a system against perturbations in its environment [18], low temperatures [19] or desiccation [20].

The occurrence of coupled folding and binding challenges us to consider the mechanism that causes it [10]: is it ‘induced fit’ (the binding partner induces the disordered IDP to adopt the bound conformation) or ‘conformational selection’ (the binding partner selects a binding-competent conformation from an ensemble of conformations visited in the unbound state)? Although these two descriptive mechanisms may not be mutually exclusive, and most likely represent extremes of a continuum [21], they emphasise the importance of the unbound ensemble [22]. However, characterising all the conformations belonging to the unbound ensemble of the IDP is challenging, because experimental techniques [23], [24], [25] generally provide only ensemble-averaged properties [5]. Since the unbound state samples a very heterogeneous set of conformations, and is likely to interconvert rapidly between them, experimental observations can be hard to interpret.

Molecular simulations can therefore play an important role in the understanding of IDP behaviour, as they provide a detailed picture of the dynamics in unfolded proteins. Phenomenological “coarse-grained” models using a simplified representation of the system are a powerful tool for investigating general features of coupled folding-binding due to their low computational cost [26], [13], [27]. However, the most detailed and accurate computational models for proteins are all-atom simulations in which all of the protein atoms and solvent molecules are included explicitly [28]. Such models should capture sequence-specific and solvent-mediated effects in a more predictive fashion. There are two main limitations of this type of simulation approach: the first is that it becomes very computationally demanding even for relatively small proteins. Enhanced sampling techniques [29], [30] such as the replica-exchange molecular dynamics (REMD) [31], [32] method used here, can help overcome this difficulty. Recent studies have used all-atom simulations to investigate the conformational dynamics of histone tails [33] and the binding of a small IDP [34].

The second limitation is that atomistic simulations represent a system as a collection of classical particles interacting via an empirical energy function or “force field” [35]. This picture is only an approximation of nature, and so the force field itself can be only approximately correct. For example, most force fields only include pairwise interactions, and neglect effects such as polarisation and charge transfer. This leads to the consequence (familiar in all coarse-graining problems) that the force field may not be fully transferable: a force field that has been parametrised with data from small molecules may suffer from flaws if it is applied to a large molecule [36], and a force field that is optimised for folded proteins may be less appropriate for IDPs.

One of the most extensively characterised IDPs is NCBD (also known as IBiD or SID [37], [38]), the nuclear coactivator binding domain of the transcriptional coactivator CBP (CREB-binding protein), which is a regulator of gene expression in animals. The domain is disordered in isolation—it forms a molten globule with some helical structure—but it undergoes synergistic folding and binding with another intrinsically disordered protein, CABD (the coactivator binding domain of the p160 transcriptional coactivator ACTR) [39], [40], [41]. NCBD also forms a complex with the transcription factor IRF-3 [42], [43], [44], which is not an IDP [45]. Each of the bound conformations of NCBD contains three helices, but the tertiary structures differ. A study of unbound NCBD [46] has found that the ensemble of the molten globule includes a ‘core’ conformer, which resembles the conformation of NCBD bound to ACTR. This experimental evidence can therefore be compared with simulation results.

In the present work, we investigate the unbound ensemble of NCBD using extensive atomistic REMD simulations in explicit solvent. Unlike recent computational studies of NCBD [47], [27], our approach is intended to be predictive: the force field is not biased towards a particular tertiary structure, and the simulation does not start from the native structure. Other recent atomistic simulations of IDPs have investigated smaller fragments using replica-exchange molecular dynamics [33] and multicanonical molecular dynamics [34] to enhance sampling. These earlier studies provide contrasting examples of the degree of secondary structure formation in the unbound state. We use an atomistic force field [48] that has been parametrised with experimental data for weakly structured peptides, which should make it appropriate for this system. By looking at the unfolded ensemble, we can reach some conclusions about how unfolded NCBD may reflect the demands of binding with ACTR. Ultimately, however, a definitive description of the binding mechanism will require explicit consideration of the binding partner.

Our simulations reveal a large amount of residual secondary structure in the unbound ensemble, in agreement with experimental evidence. The regions of NCBD that have strong 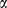-helical propensity correspond to the three helices in the experimental ‘core’ conformer of the unbound state. However, no member of the simulated ensemble has an overall structure closely resembling the core conformer, or either of the bound conformations. We find that the two (end) helices that most stabilise NCBD-ACTR binding are formed most readily. The middle helix, which is less important for binding ACTR, but which is implicated in most of the intraprotein contacts in the ACTR-bound conformation of NCBD, is formed more rarely, and tends not to coexist with the end helices. We argue that this may indicate a ‘binding interface preference’ mechanism by which NCBD retains the advantages of being disordered, while forming binding-competent structures in the unbound state. As such NCBD exhibits features of both conformational selection (with respect to the binding interface) and induced fit (with respect to contacts involved in folding).

## Results/Discussion

We have performed large-scale temperature replica-exchange molecular dynamics (REMD) [31], [32] simulations of the unbound state of NCBD (59 residues), in order to characterise the extent and nature of structure formation. REMD has proved very useful in sampling protein folding and dynamics for small proteins in explicit [49] and implicit [50] solvent. In replica exchange methods, a number of different copies (replicas) of the system are simulated in parallel, each with a different value of some parameter, in this case, the temperature. Replicas evolve independently most of the time, but pairs of replicas occasionally have the opportunity to exchange conformations. This move has a Monte Carlo acceptance probability, which ensures that each replica, given enough time, samples the canonical equilibrium at its temperature. The advantage of REMD is that higher-temperature replicas are able to cross barriers more easily, which should allow the system to reach equilibrium much more quickly.

So as not to bias the study towards any preconceived idea of what the structure should look like, our simulations were initiated from conformations that we extracted from high-temperature runs, containing little secondary or tertiary structure. The simulations used a transferable atomistic force field (Amber ff03w [51], [48], [52]), which does not incorporate any knowledge of the folded conformations. The 48-replica simulations were run for 0.25 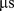 per replica, for an aggregate 12.0 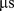 of simulation. Below, we describe the analysis of the resulting ensemble of unbound conformations.

### Residual secondary structure

**Figure 1:**
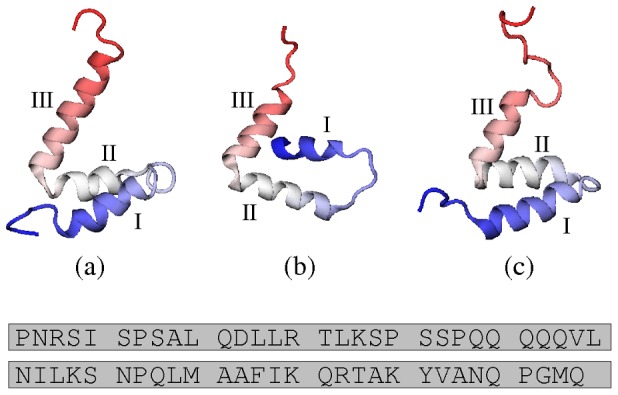
PDB structures. (a) Model 1 from 1KBH, the structure of NCBD in complex with ACTR [40]; (b) Chain C from 1ZOQ, the structure of NCBD in complex with IRF-3 [43] (the asymmetric unit comprises two NCBD molecules and two IRF-3 molecules); (c) Model 1 from 2KKJ, the core unbound conformer of NCBD [46]. In this work, we choose these conformations to represent the three PDB structures. Helical regions I, II and III are indicated for each structure. Beneath is the amino acid sequence of NCBD. We follow reference [46] in numbering the residues of NCBD sequentially from 1 to 59. This corresponds to residues 2059–2117 of mouse CBP in 2KKJ and 1KBH (also numbered 48–106 in 1KBH). Structure 1ZOQ contains residues 2065–2111 of human CBP, whose sequence matches residues 2066–2112 of 2KKJ and 1KBH; therefore, 1ZOQ contains residues 8–54 according to the numbering system that we use.

The structures which NCBD has been found experimentally to adopt in complex with ACTR (PDB ID: 1KBH) [40] and IRF-3 (PDB ID: 1ZOQ) [43] are largely 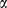-helical: both contain approximately the same three helical regions. The main difference between the two structures is in the tertiary arrangement of the helices. An experimental study [46] has found that a ‘core’ conformer (PDB ID: 2KKJ), with a structure similar to the ACTR-bound conformation 1KBH, is highly populated in the unbound ensemble. The three structures are shown in Figure 1, together with the sequence of NCBD.

**Figure 2:**
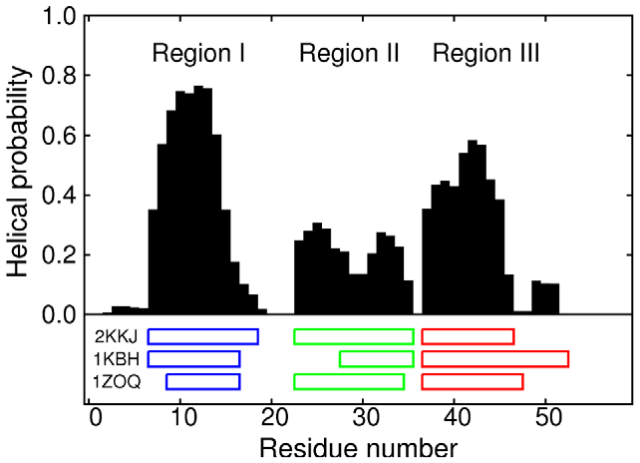
Helix propensity, expressed as the probability of a given residue being part of a sequence of three consecutive residues whose dihedral angles place them in the 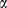 **-helical region of conformational space.** The helical region is defined by 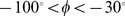 and 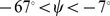 [70], [36]. The positions of the helices in three PDB structures are indicated beneath the graph: 2KKJ (the core conformer of the unfolded ensemble [46]), 1KBH (the ACTR-bound structure [40]) and 1ZOQ (the IRF-3-bound structure [43]).

In order to compare the secondary structure of our simulated unbound ensemble, we have analysed the 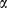-helix propensity in the 304 K replica from the REMD simulations (this is the replica whose temperature is closest to that at which the 2KKJ structure was determined [46]). Recent atomistic studies of smaller IDPs [33], [34] have shown evidence of secondary structure preference. Figure 2 shows the proportion of time spent by each residue of NCBD in a helical state; the helices in the PDB structures are also indicated.

The simulations produced three clear regions of high helical propensity, corresponding exactly to the three helices in the core unbound conformer 2KKJ: residues 7–18 (hereafter referred to as region I), 23–35 (region II) and 37–46 (region III). These results agree with the experimental observation that NCBD is intrinsically unfolded, but has residual helical structure. The helical content appears to be qualitatively consistent with CD and NMR data (discussed further below), which show a very significant helical content in the unbound state [40], [53]. The simulations also correctly predict the location of the residual helical structure within the sequence, corresponding to the regions occupied by helices in the core unbound conformer [46]. These boundaries of the helices are also in agreement with chemical shift data [53]. The main ways in which the core conformer differs from 1KBH are: (i) helix II is lengthened to cover residues 23–27, and (ii) helix III is shortened by six residues at the C-terminal end. This successful prediction provides support for the accuracy of the ff03w force field.

Our results may also show traces of the differences between the three PDB structures: the last two residues of region I (17 and 18), which are helical in 2KKJ and 1KBH but not in 1ZOQ, have lower helix propensity than the rest of helix I, while the simulations indicate a detached slightly helical region (residues 49–51) that seems to suggest the longer helix III in 1KBH, compared to the other structures; this C-terminal extension of helix III is presumably stabilised by specific interactions with the binding partner in that structure.

A recent simulation study [54] of NCBD included an REMD simulation of the unbound state, using a 47 residue fragment (corresponding to residues 8–54 of the present study) with an implicit solvent model. The authors argued that the results were limited by convergence problems; nevertheless, a simulation initiated from a fully unfolded state developed some helical propensity in regions II and III (and in region I, to a small extent). When the simulation was initiated from the folded state 1KBH, high helical propensity was found in all regions—including the part of region II (residues 23–27) that is not helical in 1KBH. This is consistent with our own findings for this portion of the sequence. Structure formation has also been seen in previous all-atom simulations of “disordered” proteins. For example, in simulations of fragments of histone tails, flickering elements of both 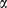-helix and 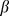-hairpin were observed [33]; similarly, a recent computational study of neural restrictive silencer factor (NRSF), which is helical when bound to its partner mSin3, was found to populate significantly both 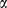 and 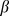 structure [34]. However, the population of helical structures is particularly high in NCBD, in comparison with these other studies. This may be related to the fact that it needs to bind another IDP, ACTR, in contrast with some other IDPs like NRSF that bind to folded domains.

In addition to individual residues, we can look at larger sections of the protein, and compute how far they deviate from some ordered reference structure, in order to evaluate the extent to which the ordered structure is present in the unbound ensemble. It makes most sense to look at regions I, II and III; since they match the helices in the 2KKJ structure, we will use that structure as the reference. The top left panel of Figure 2 shows the distribution of the root-mean-squared deviations of the individual regions at 304 K, calculated by separate least-squares fitting of each region to the 2KKJ reference structure. In this case a low RMSD is already quite a strict definition of helix formation, as it will not include short partial helices. Since the observed structures are predominantly helical, this seems like a reasonable choice of order parameters. Helix III is predicted to be the most likely to form completely (36.8% within 0.3 nm). Helix I is a little less likely to be fully formed (26.6%), probably because it is slightly longer. Helix II is significantly less stable than the others (6.15%), in agreement with the helical propensity results in Figure 2.

**Figure 3:**
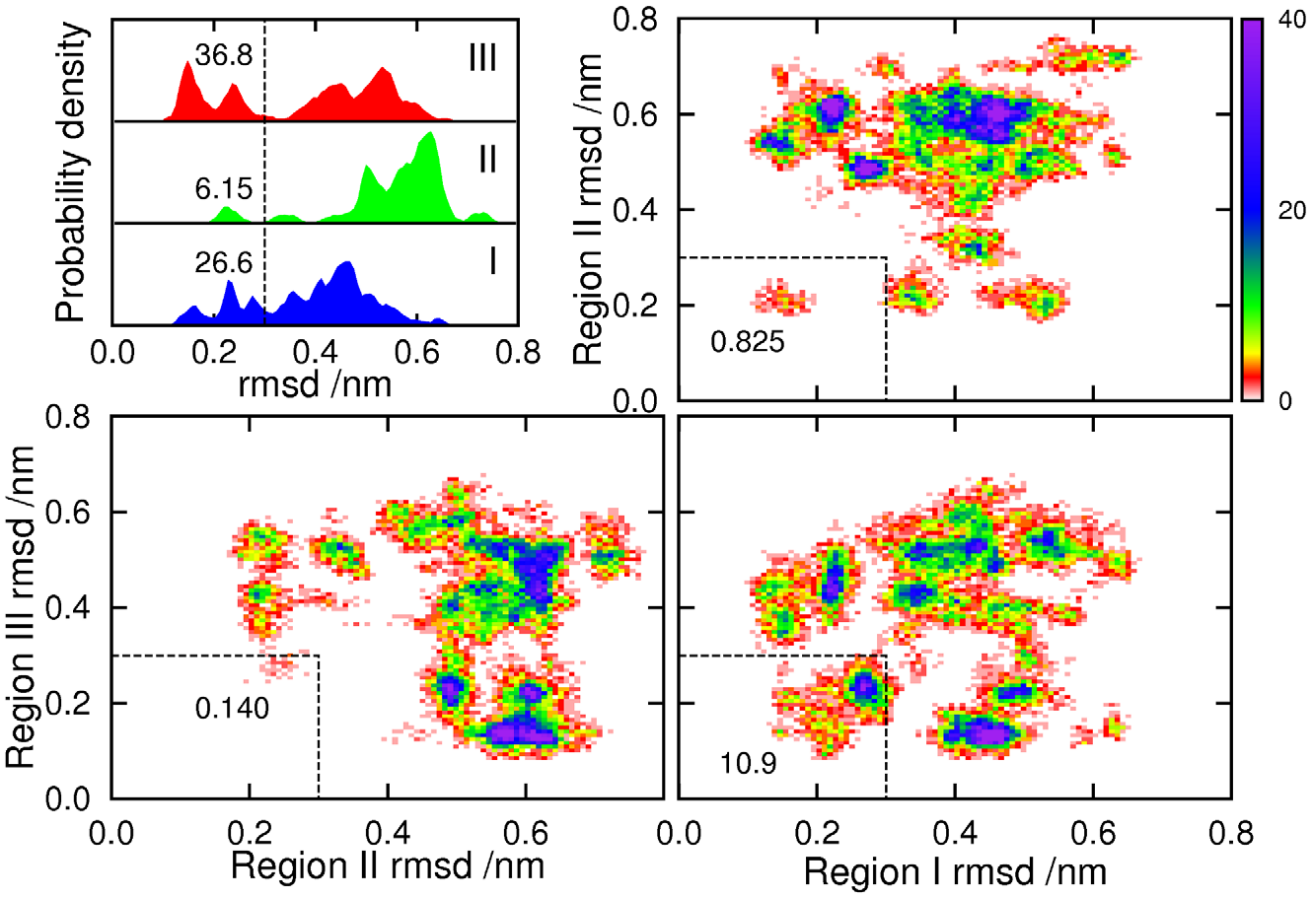
Distribution of root-mean-squared deviations of regions I, II and III, from the 2KKJ conformation. The top left panel shows the probability distributions for the three regions individually. Probabilities that rmsds are below 0.3 nm (vertical dashed line) are given as percentages. Remaining panels show the two-dimensional distributions of pairs of regions together; probabilities that both rmsds are below 0.3 nm (area bounded by dashed lines) are given as percentages.

The other three panels of Figure 3 show two-dimensional distributions of the rmsds of pairs of regions, which allow us to see the extent to which helices coexist. The darker areas, corresponding to high probability density, can be viewed as clusters of heavily populated states. The results indicate that the most common pair of coexisting helices is I and III. The joint probability that both regions are within 0.3 nm of the 2KKJ structure, at any given time, is 10.9%. This is close to the product of separate probabilities for the two regions (9.77%), which is the value that would be expected for the joint probability if the two regions were uncorrelated. Helix II is found much more rarely with either of the others. In particular, the joint probability for regions II and III is 0.140%, much lower than the product of separate probabilities (2.27%). The equivalent figures for helices I and II are 0.825% (joint) and 1.63% (separate).

**Figure 4:**
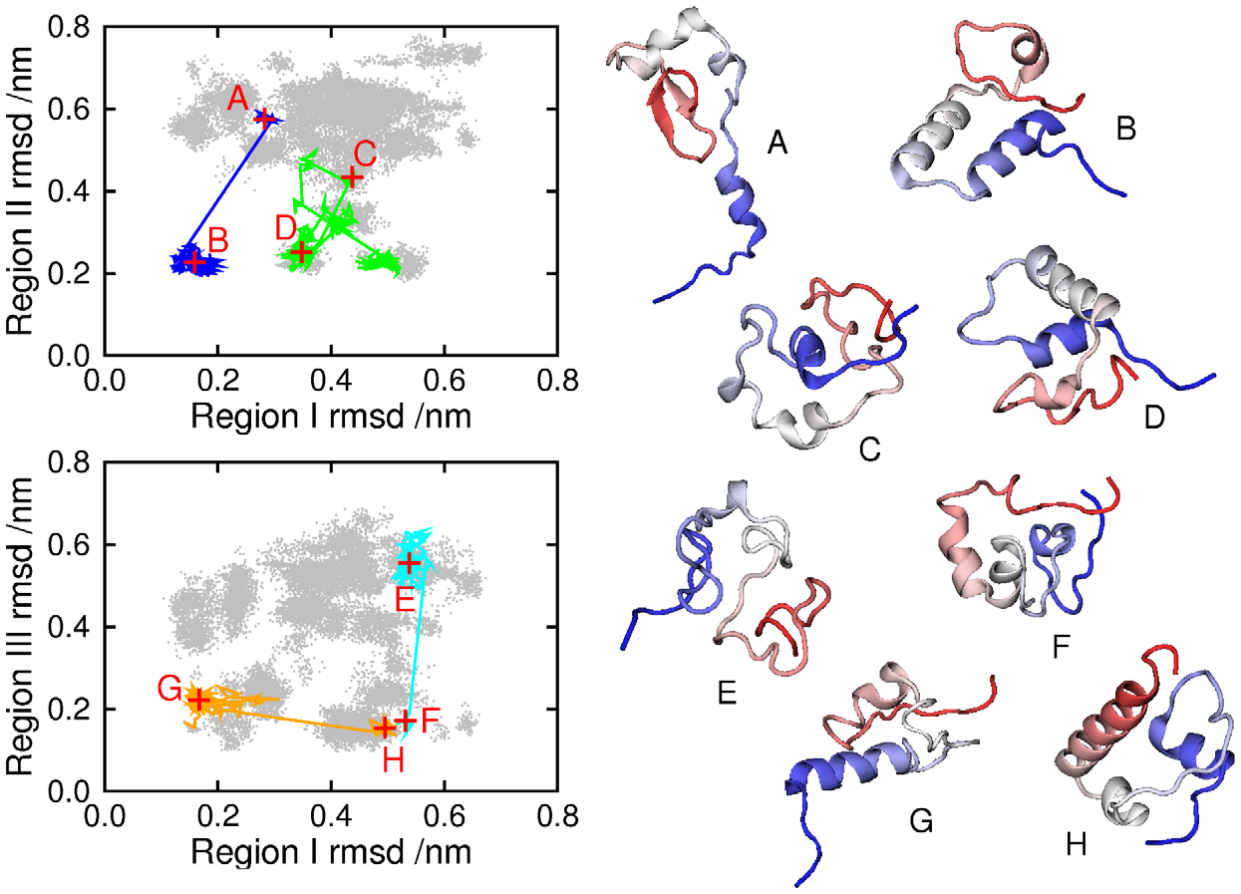
Eight conformations visited during the simulation. Panels on the left mark these in red, at positions given by the single-region rmsd measures used in Figure 2. The conformations were drawn from four continuous trajectories, also plotted on the panels (only the 304 K parts of each trajectory are shown). A: conformation at 59.36 ns (trajectory 13); B: 178.08 ns (trajectory 13); C: 159.73 ns (trajectory 35); D: 180.67 ns (trajectory 35); E: 72.98 ns (trajectory 37); F: 236.63 ns (trajectory 37); G: 60.39 ns (trajectory 42); H: 215.58 ns (trajectory 42).

One question which arises regarding any molecular simulation is how representative is the sampling. We need to be sure that the sub-ensembles which are shown in Figure 3 arise from genuine attractors in the free energy landscape of the protein, and are not a consequence of an inadequate exploration of conformational space. Evidence for adequate sampling is that free energy surfaces and helix populations calculated from the first and second halves of the trajectory (excluding the first 50 ns) produced comparable results (Figure S1). To be sure that this does not arise from configurations merely being “stuck” in local traps for the duration of the run, in Figure 4 we show eight conformations observed during the simulation, and we also mark their positions, and the continuous trajectory of which each forms a part, on a shadow of Figure 3. Only the 304 K parts of the trajectories are plotted, so each trajectory is divided into regions, joined by lines which bypass the states visited at higher temperatures. It is clear that each trajectory was able to explore a number of different regions, and also that a given region could be visited by more than one trajectory.

The trajectories in Figure 4 must not be interpreted as folding paths, since REMD aims to reproduce the canonical distribution of a system, rather than its dynamics. However, we need to inspect the trajectories, to check that they encompass changes in secondary structure. Trajectory 13 (shown in blue) forms helices I and II between conformations A (which includes a 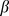 hairpin in place of helix III) and B, while helix III remains unformed. Helix III is formed in trajectory 37 (cyan) between E and F, the other two helices remaining unformed. Helix I unfolds in trajectory 42 (orange) between G and H; helix III is present, and helix II absent, throughout. Trajectory 35 (C and D), in green, illustrates multiple transitions between clusters, and the folding and unfolding of helix II.

### Maintaining intrinsic disorder

**Figure 5:**
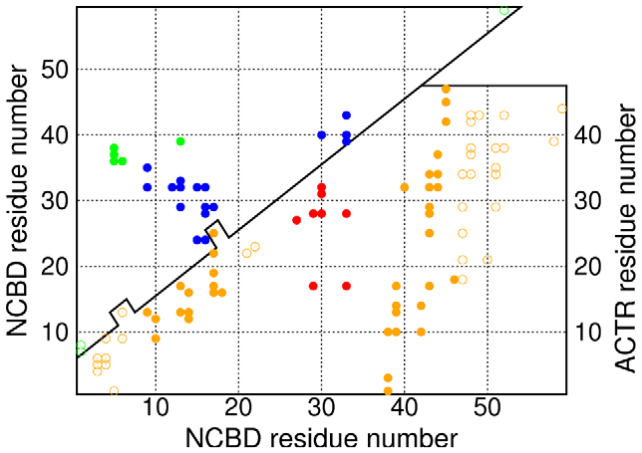
Contacts in 1KBH structure: NCBD bound with ACTR [**40**] **.** The top panel contains intra-protein contacts of NCBD: contacts shown in blue involve helix II, while contacts in green do not. The bottom panel contains the inter-protein contacts. Contacts shown in red involve helix II of NCBD, while those in solid orange involve helix I or helix III. Contacts shown in outline involve only non-helical regions of NCBD. A pair of residues is considered to be in contact if any pair of non-hydrogen atoms from the two residues are within 4.5 Å of one another.

To see more of what the simulation results can tell us about NCBD as an intrinsically disordered protein, and NCBD-ACTR binding, we look at the contacts that are present in the bound structure 1KBH. Figure 5 shows the intraprotein (within NCBD) and interprotein (between NCBD and ACTR) contacts. Of the 25 long-range (greater than 5 residues apart in sequence) intra-protein contacts, 17 involve helix II, while only five link helix III to helix I or to the N-terminal region of the protein. Turning to the inter-protein contacts, we see that only eight of the 76 contacts involve helix II (there are two more that involve nonhelical residues close to helix II). Most of the inter-protein contacts involve helix I (14 contacts) or III (23 contacts, and an additional 18 in the region 47–53, which is helical in 1KBH but not in 2KKJ).

A protein that exploits different conformations to bind different partners, as NCBD does, may face a difficulty. On the one hand, binding might be helped by the presence, in the unbound ensemble, of nativelike structure in regions of the protein that are involved in binding [55] (a recent simulation study of fast-folding proteins found that formation of secondary structure tends to precede formation of contacts [56]). This is likely to be particularly important when the ligand is itself an IDP (as ACTR is): we might expect the binding of two IDPs to be more difficult, if each spends too much of its time in conformations that are incompatible with the other protein. On the other hand, if there is too much nativelike structure, it might actually slow the binding [57], or get in the way of binding another ligand such as IRF-3.

Our results suggest that the unbound ensemble of NCBD may show adaptations that ameliorate this difficulty by means of a ‘binding interface preference’ mechanism. The regions around helices I and III contain most of the inter-protein contacts in the NCBD-ACTR bound structure, so binding might be easier if one (or both) of these regions has nativelike structure in unbound NCBD. Accordingly, the simulation results show a high degree of helicity in these regions (Figure 2; top left panel of Figure 3), and helices I and III readily occur together (bottom right panel of Figure 3). On the other hand, most of the intraprotein contacts involve helix II: a lack of nativelike structure in this region, and a tendency to avoid conformations containing both helix II and another helix, might help to make NCBD an IDP rather than a folded protein, and protect its ability to bind IRF-3. We do indeed find that helical structure is scarcer in this region (Figure 2; top left panel of Figure 3), while helix II coexists rarely with helix I, and even more rarely with helix III, which is the helix formed most commonly (top right and bottom left panels of Figure 3).

‘Binding interface preference’ is a hypothesis about the unbound ensemble, suggesting how it might be distributed in order to facilitate binding without compromising disorder in the unbound state. It is a statistical rather than a mechanistic hypothesis, and does not propose a specific binding path—indeed, it might be misleading to infer a binding path from information about the unbound ensemble alone. Unbound NCBD retains some of the structure of the ACTR-bound conformer [46]; this supports the idea that, on average, preformed nativelike structure is likely to help binding, especially when the binding partner is also an IDP. However, individual binding events might follow many different paths, not all of which need benefit from preformed nativelike structure.

**Figure 6:**
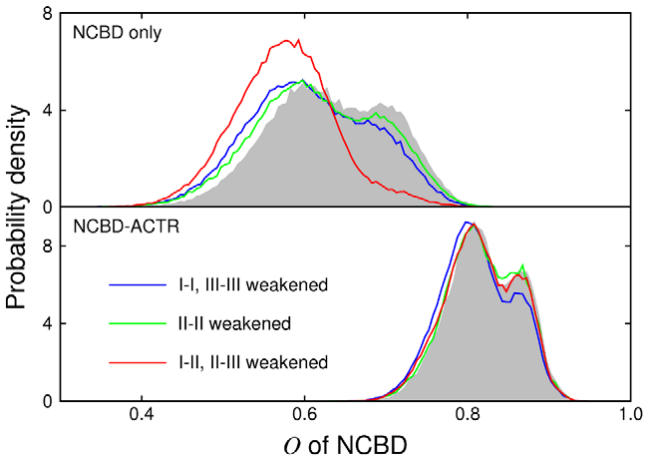
Probability distribution for 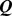 **of NCBD, computed with a G** 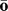 **model.** The top panel shows results for isolated NCBD, while the bottom panel shows results for NCBD in the presence of ACTR. Grey shadow: baseline model; blue lines: weakened intrahelical interactions in helices I and III; green lines: weakened intrahelical interactions in helix II; red lines: weakened interactions between helix II and the other helices.

Experimental studies of ExsE [58] and of the preS1 surface antigen of hepatitis B virus [59] suggest that, in the unbound state of some IDPs, the binding interface is more strongly structured than other parts of the protein. We can establish some further theoretical support for binding interface preference, using a coarse-grained native-centric (G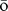) model for the NCBD-ACTR complex. Figure 6 shows the probability distribution for a nativeness parameter 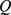, for NCBD alone and in the presence of ACTR. The model for NCBD is identical in the two cases. Because of its additional (intermolecular) interactions, the native state of the complex is more energetically favourable than that of isolated NCBD; this pushes 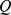 to higher values. Results are shown for a baseline version of the model, together with three variations in which subsets of the native interactions are weakened. When the interactions between helix II and the other two helices are weakened, the distribution for solitary NCBD shifts to lower values of 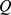 (red line); weakening the intrahelical interactions has a smaller effect (blue and green lines). This supports the idea that the disordered character of isolated NCBD is enhanced by a tendency to avoid the simultaneous formation of helix II and another helix. In the presence of ACTR, the 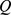 distribution is perturbed most readily by weakening of the intrahelical interactions of helices I and III, which form the binding interface. This result ties in with the idea that helical structure in these regions is particularly important for binding.

### Tertiary structure

**Figure 7:**
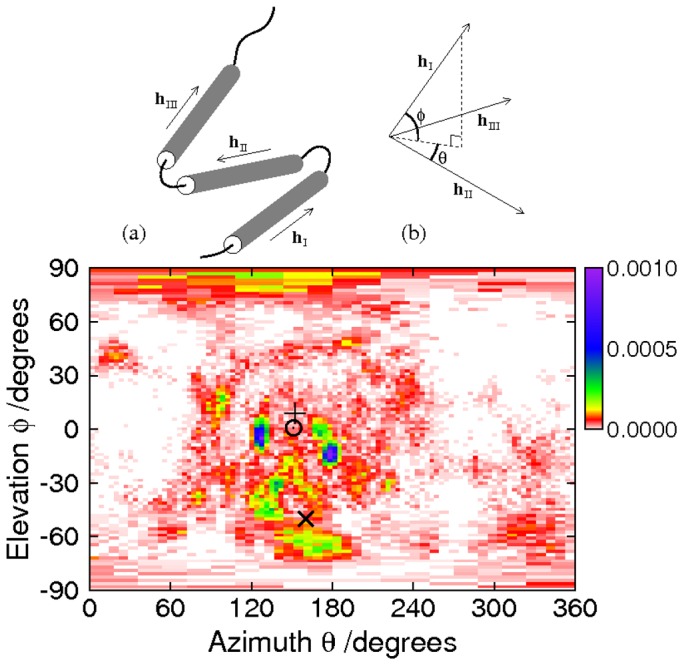
Tertiary structure in NCBD. Cartoon (a) shows how the vectors relate to the molecule: unit vectors 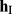, 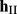 and 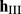 follow the principal axes of the 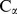 atoms in each region, whether or not helices are formed. Cartoon (b) shows how the coordinate system is derived from the vectors. A plane is defined by its perpendicular 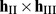. The elevation of 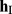 above this plane, deemed to be positive in the direction of the perpendicular, is denoted by 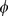, while 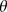 is the azimuth of 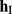, with the reference direction being 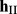 and with positive sense given by rotation from 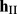 towards 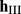 by the shortest route. The main panel shows the probability distribution of tertiary structure as a function of 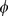 and 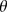. The ACTR-bound structure is marked by +, the IRF-3-bound structure by 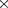, and the core unfolded conformation by 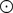. Since 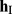 points to the surface of a unit sphere, the bins cover a larger range of 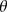 at high and low 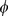, to maintain approximately constant bin size. This leads to a stretched appearance for these bins in this equirectangular projection.

Each of the bound structures of NCBD contains three helices packed together. To visualise the difference between the two tertiary structures (Figure 1), imagine a plane formed by helices II and III. Helix I can take up either of two positions: above the plane (1KBH) or below it (1ZOQ). Whether or not the helices are actually present, we can describe the tertiary structure in the coordinate system defined in parts (a) and (b) of Figure 7, which considers the orientation of region I relative to the plane formed by regions II and III. This is calculated from the relative orientations of the regions, rather than their relative positions. With only two coordinates, it cannot be a complete description of the relative orientations: it neglects the angle between regions II and III. Therefore, it is only one of many possible descriptions of the tertiary structure. However, the coordinate system is particularly appropriate for the tertiary structure of NCBD, as it captures the essential difference between 1KBH and 1ZOQ as a difference between positive and negative values of 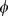.

The main panel of Figure 7 shows the probability distribution of the simulation results at 304 K, as a function of 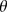 and 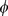. There is a favoured region on this graph, in which the three PDB structures also lie. As expected, we find that the simulated system spent more time close to the core conformer 2KKJ and the ACTR-bound structure 1KBH than in the vicinity of the IRF3-bound structure 1ZOQ. Notably, however, we observe a significant population of conformations whose topology is similar to that of 1ZOQ. The peaks in the probability do not coincide precisely with the PDB structures: the simulated ensemble does not match the secondary structures of the PDB conformations exactly (Figures 3 and 1), and therefore it lacks the specific packing interactions that would be needed to stabilise their tertiary structures; moreover, the presence of the binding partners would be expected to have an effect on the energy landscape.

The azimuth of the favoured region is closer to 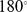 than to 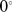, which means that the orientation of region I tends to be approximately opposite to that of region II. This orientation maximises packing interactions between helix I and each of the other helices: since all the helical regions are approximately the same length, an azimuth of 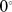 (I and II pointing in the same direction) would make interaction between helices I and III difficult. Interestingly, both in the simulations and in the experimental structures, the azimuth shows a bias towards angles below, rather than above, 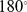, while the elevation shows a bias towards negative angles. Since there is no geometrical reason for such preferences, they can only be due to specific tertiary packing interactions.

### Comparison with experimental data

**Figure 8:**
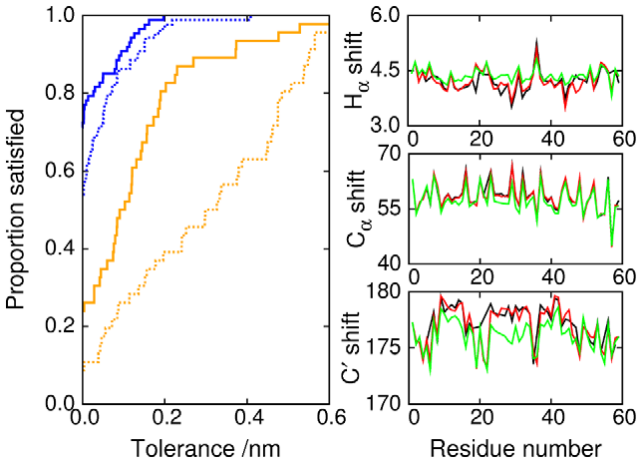
Comparison with NMR data. (Left panel) Proportion of NMR constraints satisfied (within a certain tolerance), as a function of tolerance. Blue lines represent medium range constraints (2–4 residues); orange lines represent long range constraints (greater than 4 residues). In each case, the simulation results (solid lines) can be compared with the initial randomised set of conformations (dotted lines). (Right panels) Chemical shifts calculated with SPARTA+ from the simulations (green) and the 2KKJ structure (red) are compared with the experimental values (black) [46].

To assess how close our computed ensemble is to that observed experimentally, we have back-calculated several sets of experimental data from our simulations. First, we compare our results with NMR data reported for unbound NCBD [46]. The distance restraints derived from NOE data in the experimental study were largely satisfied or near-satisfied by the 2KKJ conformation [46], which led to the proposal that 2KKJ is a ‘core’ conformer in the unbound ensemble. We compare our simulation results against these NMR restraints. The left hand panel of Figure 8 shows the proportion of the restraints that are satisfied, as a function of the tolerance, in the simulations at 304 K and in the set of 48 randomised conformations from which the simulations were initialised. Distances were computed by 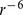 averaging over the ensemble [60]. The simulation does much better than the random set, both for medium-range constraints (between atoms separated by 2–4 residues), and for long-range constraints (separated by more than four residues). The simulation results satisfied 90.8% of the medium-range constraints within a tolerance of 1 Å (that is, allowing the simulation predictions to fall outside the range of the NMR results by 1 Å), but did less well against the long-range constraints, satisfying 50.0% within 1 Å. The remaining discrepancies are probably due to the limited sampling that was possible within the finite duration of our simulations, and to inaccuracies in the force field. Of course, the unfolded ensemble of an IDP is expected, by definition, to explore diverse conformations rather than remaining in a ‘core’ conformer or bound structure.

The right hand side of Figure 8 shows chemical shifts calculated from the simulation results using SPARTA+, and compares them with experimental values and with values similarly calculated from the 2KKJ structure. The simulation correctly reproduces the locations and extensions of the helices, as Figure 2 suggests, but underestimates the overall helical content. In earlier work, we had found that we were able to optimise the helix propensity of a simpler alanine-based sequence (the 15-residue helical peptide 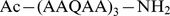), to obtain a good match to experimental chemical shift deviations [48]. The present results clearly show larger deviations from experiment, and present some measure of the transferability of the force field to more complex sequences. Some insight into this issue can be obtained from considering a more recent assessment of the helix propensities of all 20 amino acids in the ff03w force field [61]. In this work, it was found that although ff03w resulted in residue-specific propensities broadly consistent with experiment, there were significant deviations for some residues. It was also shown that optimisation of side-chain torsion potentials and the use of a common backbone charge model are promising directions for improving the helix propensity of other residues to be comparable to that of alanine [61]; thus, there is clearly further work that needs to be done to make force fields fully transferable to all residue types. We should note, however, that close to the transition midpoint, substantial population shifts can be caused by relatively small changes in free energy - e.g. for a simple two-state system a change of 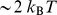 can shift the population from 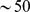
% folded to 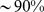 folded. Given that there are certainly appreciable residual errors in current force fields, and the challenge of sampling an equilibrium distribution for this size of system, the discrepancy from experiment in this case is in fact quite reasonable.

**Figure 9:**
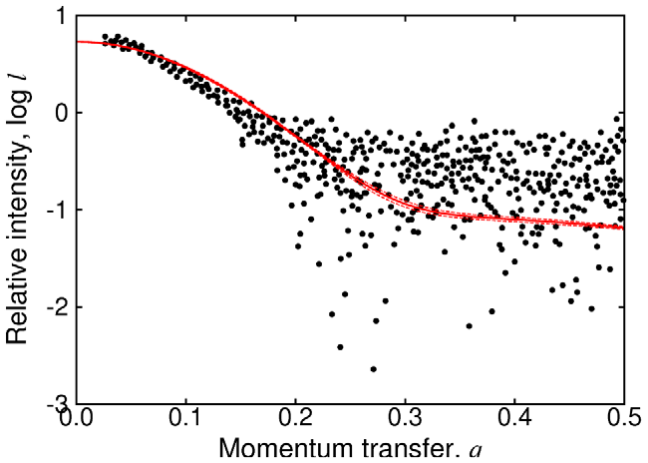
Comparison of simulation results with SAXS data. Black dots are the experimental data from reference [46]. The solid red line shows the mean of results computed from twenty randomly chosen frames of the simulation using CRYSOL [62]; dashed red lines indicate the confidence interval for the mean. Since we are interested in relative rather than absolute intensities, an offset has been added to the calculated intensities, to best fit the experimental data.

Small-angle X-ray scattering (SAXS) data contain information about the gross features of the structural ensemble. We have computed SAXS curves, using CRYSOL [62], from structures sampled randomly from the 304 K replica in our simulations. The ensemble-averaged SAXS profile is compared with the experimental data in Figure 9. The data agree reasonably well in the low-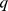 region which is most sensitive to large-lengthscale features of the ensemble. However, in this region the second derivative of the simulated intensity is less negative than that of the experimental intensity. According to the Guinier approximation [63], this indicates that the simulated ensemble for the unbound state is slightly compacted relative to the experimental system. This is consistent with the average radius of gyration of 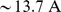 estimated from the simulations using CRYSOL. The comparable figure estimated from the unbound 2KKJ structure is 15.8 Å, while the average value reported from SAXS experiments under nativelike conditions is 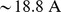 [46]. Overcompaction of unfolded proteins is a common problem with current force fields. Although previous studies with the TIP4P/2005 water model [48] or TIP4P-Ew (a water model similar to TIP4P/2005) [64] gave more realistic dimensions for the unfolded state than the commonly used TIP3P water, it is clear that there is room for further improvement.

### Conclusions

The simulation results successfully reproduce the essential characteristics of unbound NCBD: it is found to be a molten globule with residual 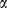-helical structure. In respect of secondary structure, we find very good agreement between the simulations and the experimentally observed core unfolded conformer 2KKJ. This agreement can be seen in the proportion of medium-range NMR restraints satisfied, and in the residual helical propensity. Importantly, the helical propensity matches the core conformer very closely indeed, whereas it matches the bound structures of NCBD only to the extent that they resemble the core conformer. The simulations are less successful at reproducing the tertiary structures of the core conformer or of the bound structures: no single simulated conformation is a close match for any of these. However, if the tertiary structure is plotted according to a two-dimensional coordinate system that is based on the difference between the two bound structures 1KBH and 1ZOQ, the simulated ensemble is found to favour regions in the vicinity of the PDB structures.

Helices I and III of NCBD contain the bulk of the residues that are in close proximity to ACTR in the NCBD-ACTR bound structure. Therefore, it is likely that these regions are where preformed structure will be helpful for binding, particularly as ACTR itself is intrinsically disordered. In contrast, helix II is involved in most of the intraprotein contacts of NCBD. This suggests that preformed structure in the region of helix II, especially in concert with preformed structure elsewhere, might encourage the formation of the ACTR-bound structure of NCBD, even in the absence of ACTR, thereby eliminating the benefits of disorder.

In the simulations, helices I and III form more readily than does helix II, while coexistence between helix II and the other helices seems to be disfavoured. These facts point to the hypothesis that a ‘binding interface preference’ mechanism is helping to maintain disorder in the unbound state of NCBD—by disfavouring structure that might encourage independent folding—while favouring structure that facilitates binding with ACTR. On the continuum between conformational selection and induced fit, regions of NCBD that form contacts with ACTR are perhaps closer to conformational selection, while regions that form intraprotein (folding) contacts in the ACTR-bound structure are closer to induced fit. It would be interesting to see if any other IDPs, particularly ones with intrinsically disordered binding partners, use an analogous mechanism.

## Methods

The simulated system contained a single molecule of 59-residue NCBD in a truncated octahedral water box of size 70 Å between nearest faces, with periodic boundary conditions. NCBD comprised 944 atoms, and the box contained 8334 water molecules, for a total of 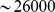 atoms, including a low (0.15 M) concentration of sodium chloride. The water model used was TIP4P/2005 [65] (a highly optimised version of TIP4P) while the Amber ff03w force field [48] was used for the protein (this force field has been adapted for use with the TIP4P/2005 water model). Long-range electrostatics were calculated using the particle mesh Ewald (PME) method, with a 9 Å cutoff and a 1.2 Å grid spacing. Simulations were performed with GROMACS 4.5.3 [66], using Phase 2b of HECToR together with in-house computing resources. We used replica-exchange molecular dynamics [32], with a total of 48 replicas. Replicas differed only in temperature: the lowest temperature was 304.000 K, the highest 424.458 K. Temperature gaps between replicas were selected to ensure an exchange acceptance probability of slightly above 0.2 between neighbouring replicas, throughout the temperature range. This led to a choice of 2.000 K for the temperature gap at the low temperature end, increasing to 3.221 K at the high temperature end.

The above system, with the protein initiated from the 2KKJ structure, was simulated for 8 ns at 304 K under conditions of constant pressure. From the resulting trajectory, a conformation was selected which had a volume close to the trajectory average. This was used as the starting point for the subsequent preparation and simulations, which were performed under conditions of constant volume. To produce 48 starting conformations for the REMD, the system was simulated at constant volume at 600 K. Conformations were selected from different points on the trajectory between 10 and 100 ns. Each conformation was then equilibrated at its appropriate starting temperature (in the range 304.000–424.458 K) over a cooling time of 50 ps. A danger of using a simulation at 600K is that the groups around peptide bonds adjacent to proline residues may isomerise, and be trapped in the incorrect isomer on cooling to the simulation temperature. Therefore, care was taken to select conformations from points on the trajectory where only the correct isomers were present.

After this preparation, the replica-exchange molecular dynamics was run for a total of 250 ns. A different random seed was used for the Langevin dynamics of each replica. The simulation time step was 2 fs, and replica exchanges were attempted every 10 ps (which corresponds to attempting half of the possible exchanges every 5 ps). We monitored structural properties, and on this basis the first 50 ns was discarded, leaving us with 200 ns of usable data.

A practical problem with explicit solvent simulations of unfolded proteins concerns the choice of box size. Under periodic boundary conditions, it is possible for the protein molecule to interact with itself across the boundary, and possibly form a complex, which is clearly an artefact. Unfortunately, the length of time that can be simulated diminishes rapidly as the box size is increased: for a 59-residue protein such as NCBD, it is unrealistic to use a box so large that self-interaction is impossible. We can only choose a box large enough to make self-interaction a rare event. However, the closer a simulation gets to exploring a representative sample of the whole ensemble (which this study aimed to move towards), the more likely it becomes that such unfortunate rare events will happen. Artefactual complex formation occurred in two of the 48 continuous trajectories, and we disregarded all the data from these two trajectories. None of the other trajectories showed any close approaches between the molecule and its image. The minimum separation between the molecule and its image was never less than 6 Å, and was less than 10 Å only 0.2% of the time. The separation was between 20 Å and 40 Å about 98% of the time. Since close approach was such a rare event, it is unlikely that the finite box size had much influence on the ensemble (Figure S2 shows the probability density for this minimum separation).

For the G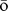 model simulations (Figure 6), models were constructed from the native state 1KBH according to the prescription of Karanicolas and Brooks [67], except that the pseudoangles between three consecutive residues were subject to a statistical potential [68], [69]. Langevin dynamics simulations were run using CHARMM, with a time step of 0.01 ps and a friction coefficient of 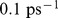. Each variation of the model was simulated for between 8.5 and 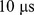, and the first 500 ns was discarded. In variants where a subset of native contacts is weakened (by a reduction of 20% in thair pair energies), the pair energies of the remaining contacts are increased so as to preserve the energy of the native state. The nativeness parameter 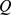 is defined, as a function of residue separations 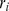, by
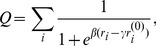
(1)where 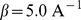 and 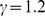. The sum is computed across all native contacts 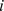, and 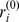 is the residue separation in the native state.

## Supporting Information

Figure S1 Convergence of results. Left panels: helix propensity (as in Figure 2). Top left: distributions calculated over 50–150 ns; bottom left: 150–250 ns. Right panels: probability distribution of rmsds of helices I and III (as in Figure 3). Top right: distributions calculated over 50–150 ns; bottom right: 150–250 ns.
(TIF)

Figure S2 Distribution of minimum separation between the molecule and its image.
(TIF)
