# Supplementary figures and images for: A Preformed Binding Interface in the Unbound Ensemble of an Intrinsically Disordered Protein: Evidence from Molecular Simulations
Source: PLoS Comput Biol. 2012 Jul 19;8(7):e1002605. doi: 10.1371/journal.pcbi.1002605 (PMC3400577; doi:10.1371/journal.pcbi.1002605)

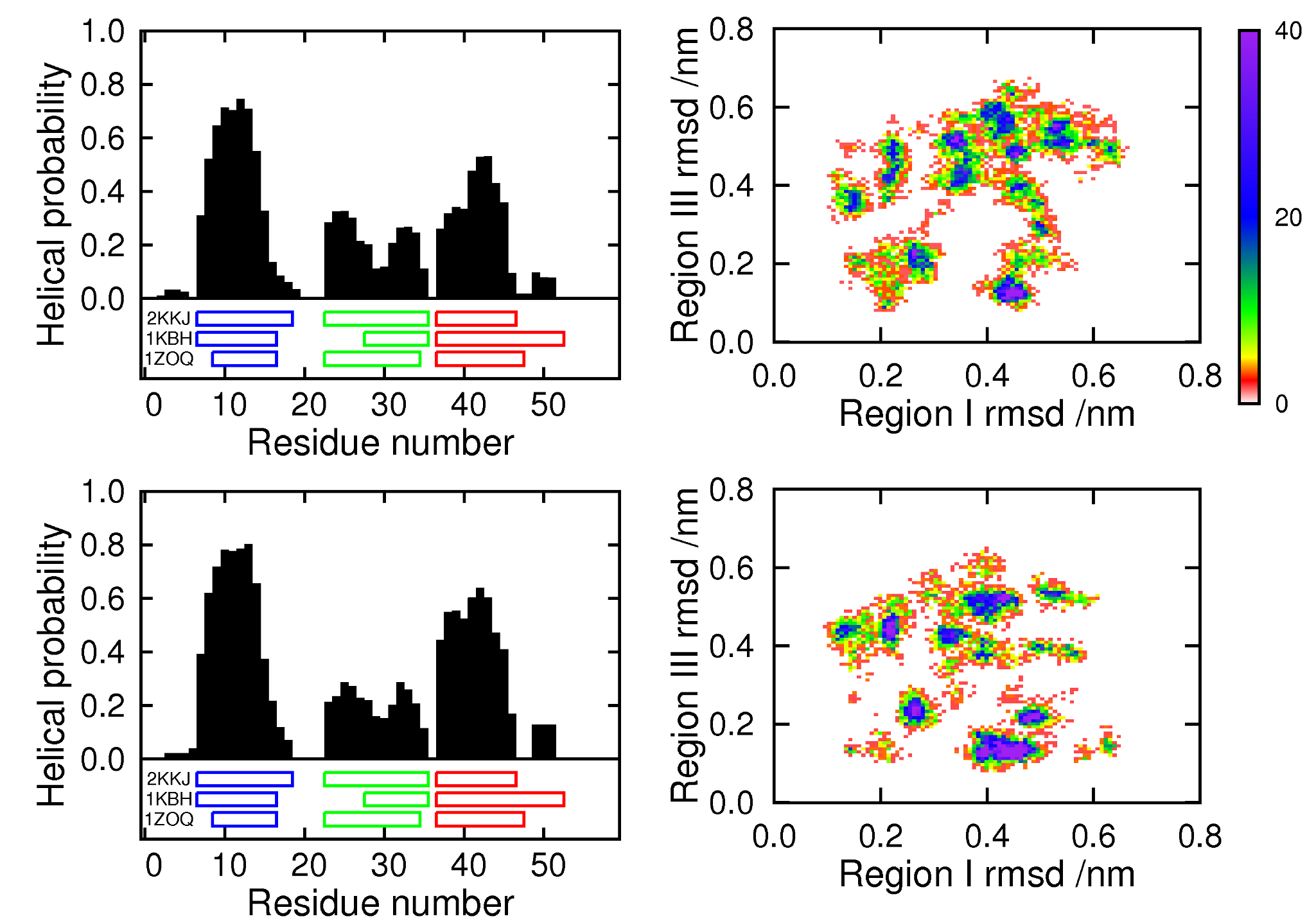

Supplement: Figure S1 — Convergence of results. Left panels: helix propensity (as in Figure 2). Top left: distributions calculated over 50–150 ns; bottom left: 150–250 ns. Right panels: probability distribution of rmsds of helices I and III (as in Figure 3). Top right: distributions calculated over 50–150 ns; bottom right: 150–250 ns. (TIF) [file pcbi.1002605.s001.tif]

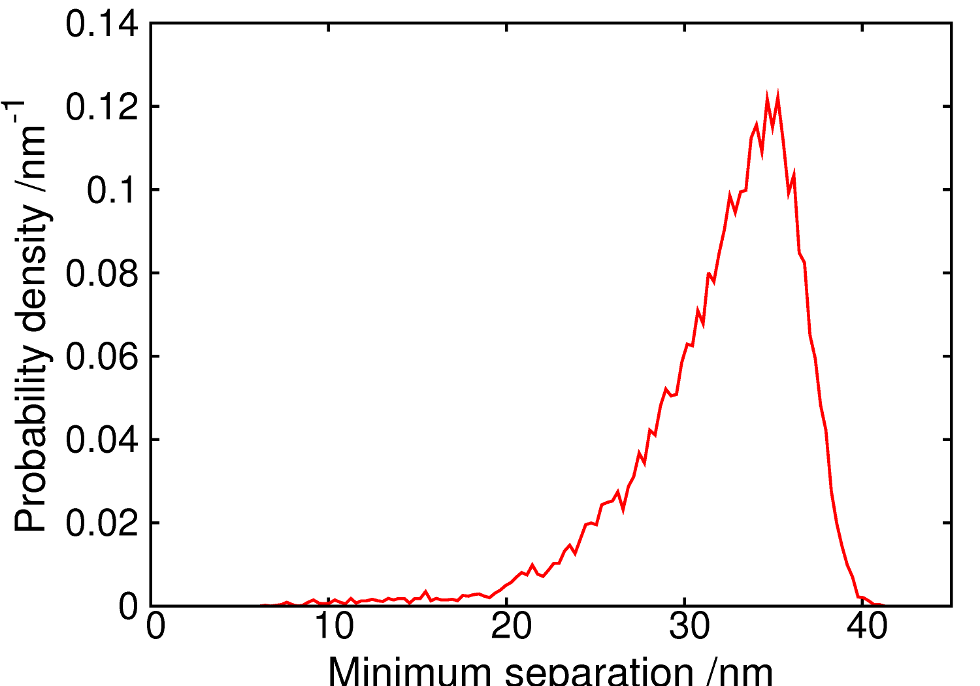

Supplement: Figure S2 — Distribution of minimum separation between the molecule and its image. (TIF) [file pcbi.1002605.s002.tif]
